# Linear mixed-effects models for within-participant psychology experiments: an introductory tutorial and free, graphical user interface (LMMgui)

**DOI:** 10.3389/fpsyg.2015.00002

**Published:** 2015-01-22

**Authors:** David A. Magezi

**Affiliations:** Neurology Unit, Laboratory for Cognitive and Neurological Sciences, Department of Medicine, Faculty of Science, University of Fribourg Fribourg, Switzerland

**Keywords:** linear mixed-effects models, experimental psychology, within-participant design, graphical user interface, R

## Abstract

Linear mixed-effects models (LMMs) are increasingly being used for data analysis in cognitive neuroscience and experimental psychology, where within-participant designs are common. The current article provides an introductory review of the use of LMMs for within-participant data analysis and describes a free, simple, graphical user interface (LMMgui). LMMgui uses the package *lme4* (Bates et al., 2014a,b) in the statistical environment R (R Core Team).

Linear mixed-effects models (LMMs) provide a versatile approach to data analysis and have been shown to be very useful in a several branches of neuroscience (Gueorguieva and Krystal, 2004; Kristensen and Hansen, 2004; Quené and van den Bergh, 2004; Baayen et al., 2008; Lazic, 2010; Judd et al., 2012; Aarts et al., 2014). The current article briefly reviews the use of LMMs for within-participant studies typical in in experimental psychology, before describing a free, graphical user interface (LMMgui; http://doi.org/10.25592/lmmgui) to carry out LMM analyses.

## Why would one use LMMs to analyse within-participant data?

Let us consider a hypothetical experiment where a researcher is interested in how quickly human listeners can detect a telephone ringing in the presence of concurrent speech. The response variable collected is the average reaction time (RT), and at first, only one explanatory variable is available: language. Measurements of RT are available for concurrent speech in French, German, and English, and thus language can be described as a categorical factor with three levels. RTs may have been measured from three different groups of monolingual listeners. Importantly, each measurement would be from a different listener. Such data is grouped by listener and by language, and since each listener can only belong to one language group, the grouping factors of listener and language are said to be nested. In this case, language can also be described as a “between-participants” factor, and the data may be analyzed with a standard analysis of variance (ANOVA). This method assumes that the response variable comes from a normally distributed population and shows homogeneity of variance.

Now it may be that the measurements were obtained in a very different manner. If the measurements came from a single group of multilingual listeners who all performed the task in each language, then language would be described as a “within-participants” factor. These measurements cannot be considered as independent because three measurements (“repeated measures”) were collected per listener. This phenomenon, which is known as pseudoreplication, is common in neuroscience experiments and leads to the use of repeated-measures (rm) ANOVAs. rmANOVAs require two additional assumptions (for example, see Maxwell and Delaney, 2004; Nimon, 2012). The first assumption is compound symmetry, which means that in addition to homogeneity of variance, the covariances are similar. Covariance appears along the off-diagonal elements of the variance-covariance matrix, while variance appears along the diagonal. In the current example, compound symmetry means that not only should the three diagonal elements (one for each level of the factor language) be similar, but so should the off-diagonal elements. If the stringent assumption of compound symmetry is violated, then sphericity must hold. Sphericity means that the variances of the difference scores (between the three levels of language) are similar. Violations of the sphericity assumption can lead to an increase in Type I errors (rejection of the null hypothesis, when it is actually true), and can be problematic for traditional *post-hoc* tests such as Tukey LSD problematic (Howell, 2009). Although sphericity is often violated in experimental psychology data sets, there are solutions for the Type I error rate (for review, see Keselman et al., 2001). One potentially conservative solution is to use the Greenhouse-Geiser or Huyn-Feldt methods to correct for degrees of freedom. An alternative solution is a multivariate ANOVA, which would require more listeners than factor levels (Oberfeld and Franke, 2012). The second assumption of rmANOVAs is complete data; for each listener, measurements must be available for all three languages. Non-completing listeners must either be excluded, or have their missing data imputed (Overall and Tonidandel, 2006).

In stark contrast to rmANOVAs, LMMs do not depend on limited assumptions about the variance-covariance matrix and can accommodate missing data. Furthermore, LMMs provide the ability to include various configurations of grouping hierarchies: multiple, nested groups such as street, town, country, and continent; partially-crossed groups, such as student and teacher in a large school where not all students interact with all teachers; and fully crossed groups. This flexibility explains social scientists increasing use of LMMs, also known as “multilevel” or hierarchically linear models. However, it is important to realize that the use of LMMs is by no means restricted to complex grouping designs, and can also be used for experimental psychology studies with a single grouping factor of participant or subject. Importantly for the experimental psychologist, LMMs also allow one to explicitly model the effect of stimulus tokens. For example, in our hypothetical experiment the concurrent speech may have been provided by different multilingual speakers. If each speaker was presented to each listener under all experimental conditions, speaker can be considered a fully crossed, within-participant random factor. A further advantage is that, in some situations, LMM results provide better interpretability in terms of physiological phenomena and a superior fit to the data (Kristensen and Hansen, 2004).

## What is an LMM?

Like many statistical models, an LMM describes the relationship between a response variable and other explanatory variables that have been obtained along with the response. In an LMM, at least one of the explanatory variables must be a categorical grouping variable that represents an experimental “unit.” In the above example, that would be an individual listener.

When using LMMs, it is important to classify explanatory variables either as “fixed factors” or “random factors.” Fixed factors are those where all levels of interest are actually included in the experiment. For example, in studies which are interested in the difference between males and females, the factor of gender with two levels would be a fixed-factor. In contrast, random factors, also commonly referred to as “grouping variables”, include only a sample of all possible levels. Although researchers are often interested in studying a large population, such as adult humans, psychology experiments typically only include a very small subset of that population, so that participant is a random factor. Classification of a factor is not always a trivial task. For example, consider the factor language in our hypothetical experiment. Do the researchers have theoretical or practical reasons to be only interested in the differences between French, German, and English specifically, or would they like to generalize their findings to all languages? In the former case, language would be a fixed factor and in the latter, a random factor. In fact, to generalize to other stimuli within a language, one should also treat the individual stimulus tokens, in our example the speaker, as a random-factor (Baayen et al., 2008; Judd et al., 2012). Hierarchical grouping factors, such as “town” or “teacher” discussed above, are often treated as random factors.

LMMs comprise two types of terms: “fixed-effects” and “random-effects,” hence the label “mixed-effects.” The fixed-effects terms comprise exclusively fixed factors, and the fixed-effect part of a LMM can vary in complexity depending on which terms are included. The “full” LMM includes the highest-order interaction between the fixed factors, as well as lower-order interaction terms and main effects, whereas other LMMs would include only some of these terms. Note that for data analysis, it is also important to distinguish between categorical fixed factors such as language or gender, which are sampled from a population of discrete levels, and continuous fixed covariates (numeric variables). An example of the latter is the sound level of the telephone in our hypothetical experiment: RTs were measured with the telephone ringing at different sound levels (60, 70, and 80 decibels sound pressure level, dB SPL), while the sound level of the concurrent speech was fixed.

**Figure 1:**
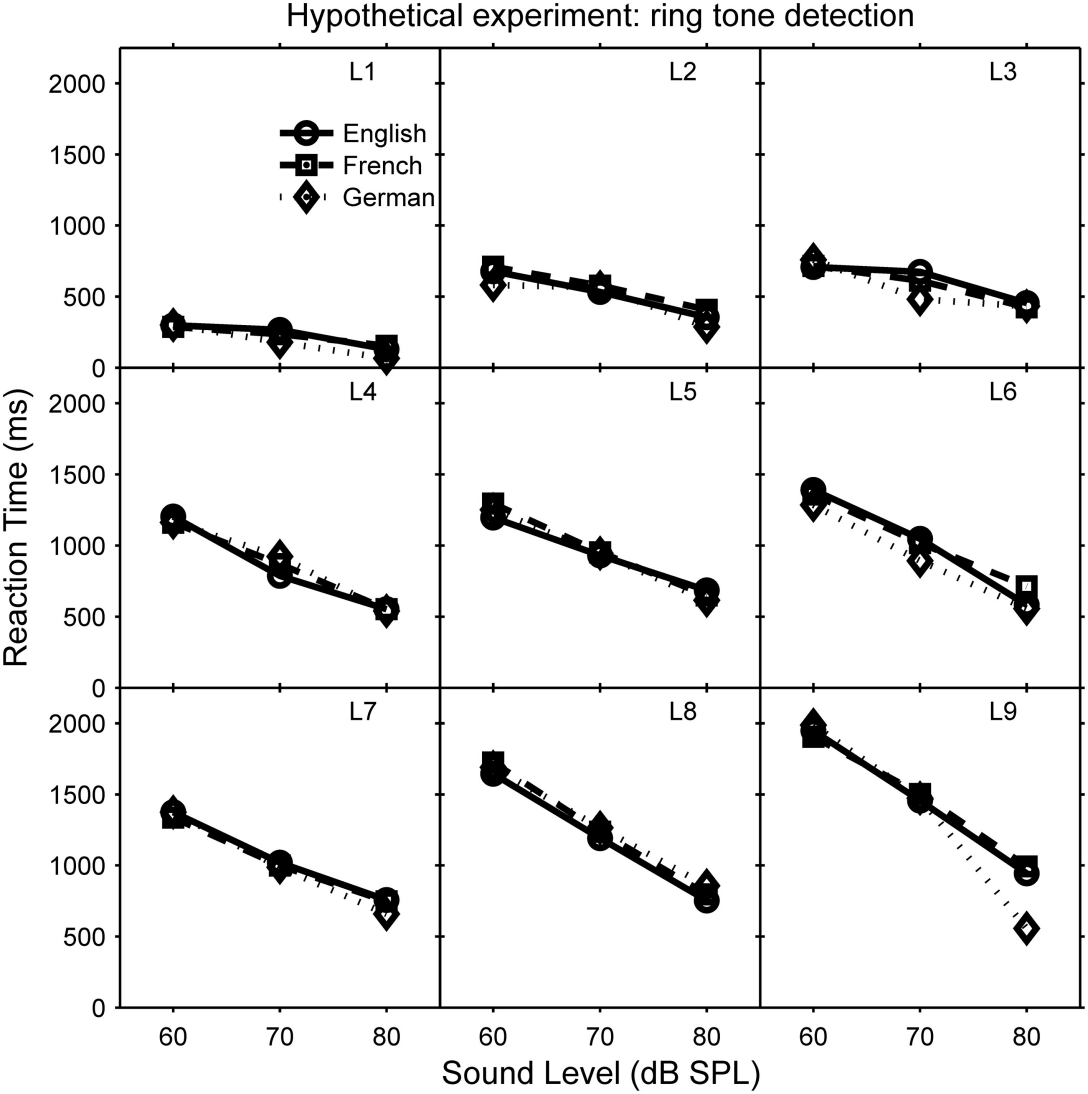
**Reaction times (RT) in ms as a function of telephone sound level in dB SPL during a hypothetical telephone-ringing detection experiment**. Each panel represents a different listener (L1–L9) and each line represents a different language of the concurrent speech (see legend in panel of L1). RT decreases with increasing sound level, and the gradient of this function (“slope”) is correlated with the RT at 0 dB (true “intercept” is not shown), which varies between listeners.

The random-effects terms of LMMs are all the terms that include random factors; interactions between fixed and random factors are considered in the random-effects terms. For example, in the hypothetical telephone-ringing experiment, the random factor listener and its interaction with the fixed covariate sound level can be modeled using a number of different random-effects terms, which differ in their complexity (number of parameters). The simplest random-effects term, known as “random intercept only,” ignores the interaction: it only considers how RT at zero sound level (0 dB SPL) varies between listeners. This is analogous to the assumption of compound symmetry. However, RT may vary as function of sound level, for example RT could decrease with increasing sound level. The slope of this function may vary between participants, and to account for this interaction between participant and sound level, we would also need to include a “random slope” term. In the full LMM, the random-effects part would also include parameters that allow for the intercept and slope to be correlated: for example, if as shown in Figure 1, listeners with a higher RT at 60 dB (higher intercept at 0 dB, which is off the displayed scale) show a greater rate of RT reduction with increasing sound level (steeper slope).

## How could one use LMMs to analyse data?

One approach to using LMMs is to systematically compare the full LMM to other models which are the same except for one term missing. The comparison is done using a likelihood-ratio test (LRT), and the test statistic χ^2^, degrees of freedom and *p-value* are reported for the missing term. A *p*-value of less than 0.05 (see below) is often considered to indicate that the missing term contributed significantly to the model fit. Care should be taken in interpreting the results because the hypothesis test involves a comparison on the boundary of possible conditions for which the χ^2^ test can be conservative (for further discussion, see Pinheiro and Bates, 2000; Bates, 2010). Some authors have argued that calculating the correct degrees of freedom is problematic and that LRTs for small group sizes (<50) lead to increased Type I error (Pinheiro and Bates, 2000). However, this has not been found to be the case in typical within-participant experimental psychology data sets where the number of measurements per participant is high relative to the number of model parameters (Baayen et al., 2008; Barr et al., 2013).

Although LMMs are useful for both confirmatory hypothesis tests and exploratory analyses, it is important to distinguish between these two when reporting results. The former are tests based on hypotheses, which were posited before data collection, and motivated the study design (Tukey, 1980). After data collection, the planned tests are performed and the test statistics and degrees of freedom are reported along with a *p*-value, which is thought to indicate the probability that the value of the test statistic or greater would have been obtained under the null hypothesis. In contrast, exploratory analyses are based on statistical tests which are motivated by the pattern of results observed after data collection. In neuroscience, there is pressure to publish studies with *p*-values below 0.05, which is often considered to be “significant,” although this pressure has often been criticized (Rosenthal and Gaito, 1963; Rosnow and Rosenthal, 1989; Nuzzo, 2014). This leads to several different exploratory analyses being performed, and when a significant result is found, this exploratory analysis is reported as if it were a confirmatory test. The result is distortions in the literature and difficulties in reproducibility of published results (Ioannidis, 2005; Simmons et al., 2011; Wagenmakers et al., 2012; Ioannidis et al., 2014).

## Software

**Figure 2:**
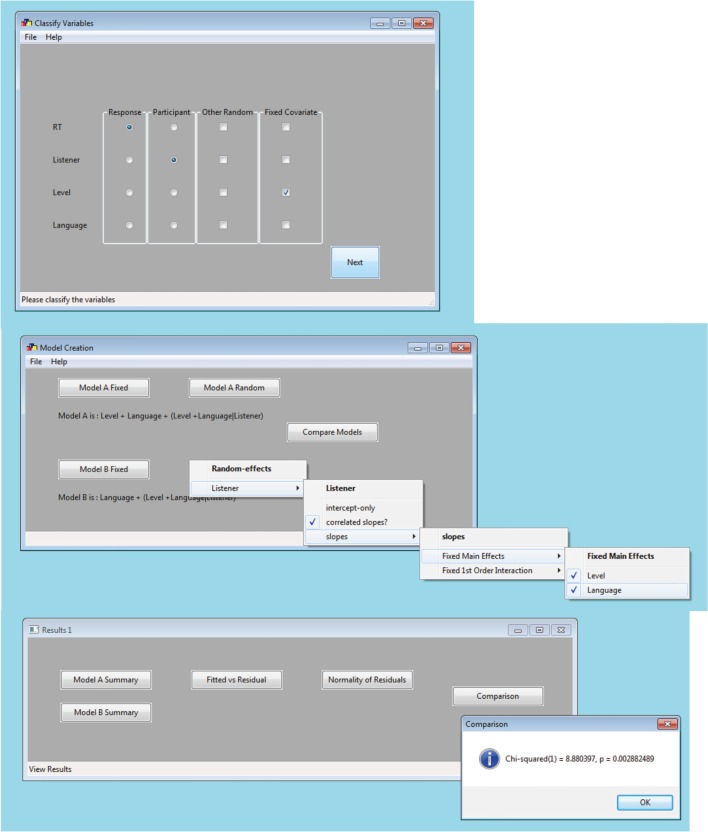
**Example windows of LMMgui**. Once a data file has been selected, the user is requested to classify the variables using the top window. In this hypothetical example (see Equation 1), the variables are classified as follows: “RT” is the response variable, “Listener” is the participant variable (random factor) and “Level” (sound level) is a continuous fixed covariate. Any variables which are not classified by radio-button or check box are treated as discrete fixed factors: in this example, “Language.” Once variables have been classified, the next step is model specification (middle window). Users select which terms to include in two models by checking items in the drop down menu. The results of the models fits and comparison are available from the results (bottom) window. The user can inspect a summary of each model, diagnostic plots (fitted vs. residual plot and histogram of normality of residuals), and the result of the model comparison (shown). File format details, prerequisites, and output files are described in the Appendix.

LMMs are available in commercial programs such as SPSS (“mixed”), SAS (“proc mixed”), S-PLUS, MLwiN, or ASReml. LMMgui, is a free, graphic user interface that uses *lme4* (Bates et al., 2014a,b), a package in the free, open-source program R (R Core Team, 2014). LMMgui is aimed at experimental psychologists who would like to use *lme4* but are not yet familiar with R and command-line programing. It provides a simple interface to classifying variables (i.e., as random or fixed factors; Figure 2 top window) and then to specify two LMMs (middle window). An LRT is used to compare the models. Details of the LMMs, diagnostic plots and the result of the LRT (χ^2^, degrees of freedom and *p*-value) are available for inspection (Figure 2, bottom window). The plots allow one to inspect for the assumptions of linearity and homoscedasticity (fitted vs. residual), as well as normality of the residuals. Interpretation of these plots, as well as model summaries is beyond the scope of this mini-review, but has been described previously (Pinheiro and Bates, 2000; Bates, 2010; Bates et al., 2014b)

For the hypothetical data shown in Figures 1, 2, the explanatory variables are: language (within-participants, categorical fixed factor), sound level (within-participants, continuous fixed covariate), and listener (participant, random factor). The random factor of speaker has been omitted for clarity. LMMgui creates models using the *lmer* function of *lme4*, and an LMM could be expressed as:

(1) RT~Language+Level+(Language+Level|Listener)

Where “RT” is the response variable and the model terms are to the right of the tilde character (“~”). The first terms are fixed-effects: “Language” and “Level.” An interaction term would include a colon “:.” The random-effects terms are those which include a bar symbol (“|”). To the right of the bar is the random factor “Listener.” The expression to the left of the bar indicates that this random term includes correlated intercepts and slopes for the fixed factors. “(Language + Level|Listener)” implicitly includes the random intercept and is equivalent to “(1 + Language + Level|Listener).” In contrast, a random-intercept only term would be “(1|Listener),” and the term for uncorrelated random intercept and slope would be “(Language + Level || Listener).” Further examples and alternative syntax for model terms are given by Bates et al. (2014b: Table 2).

In order to evaluate the main effect of level, the above model can be compared to a model without the term of interest, that is:

(2) RT~Language+(Language+Level|Listener)

Note that during evaluation of fixed-effects, it is recommended that the random-effects part of the models always includes slopes for all fixed factors because this has been shown to be important for confirmatory hypothesis testing in experimental psychology (Barr, 2013). However, such “full” random-effects terms may be inappropriate if random factors are not fully crossed, and may lead to failure of the model to converge (for discussion and possible solutions, see Barr et al., 2013).

As with most statistical analyses, an important computational step is estimating the parameters of the LMM. Although the details of this are beyond the scope of this mini-review, the reader should be aware of standard maximum likelihood (ML) and restricted ML (REML) criteria. Although the default REML may provide a better estimate of random-effects standard deviation, it does so by averaging over some of the uncertainty in the fixed-effects parameters. For this reason, the ML criterion is used when comparing LMMs with different fixed-effects structures.

A significant LRT would indicate that the missing fixed-effects term (interaction or main effect) is important. For example, the hypothetical data (Figures 1, 2) show a significant main-effect of sound level. Note that the presence of a significant higher-order interaction may make interpretation of lower-order interactions/main-effects difficult.

Although at present LMMgui is only available for continuous response variables from a normally distributed population, mixed-effects models can also be used for categorical response variables (Dixon, 2008; Jaeger, 2008). *lme4* includes the function *glmer* which can be used for count data (Poisson distribution), binary/proportion data (binomial), and for data whose variance increases with the square of the mean (gamma). Introductory books are available for further reading on the use of R in general (Crawley, 2013), and mixed-effects models in psychology (Baayen, 2008). For the reader already acquainted with the command line interface of R, there are a number of helpful packages for systematic evaluation of LMMs, such as afex, car, ez, lmerTest, pbkrtest.

## Conclusion

In order to promote simplicity of use, LMMgui is not as comprehensive as using the command-line options. It is likely that there may be some criticism for a program that provides such a simple interface; Barr et al. (2013) cite a prominent scientist who remarked that encouraging experimental psychologists to use LMMs “was like giving shotguns to toddlers.” However, many experimental psychologists already understand how to use rmANOVAs, and are capable of learning the guidelines for LMM use. It is hoped that this article and LMMgui may help them start to take their first steps in that direction.

### Conflict of interest statement

The author declares that the research was conducted in the absence of any commercial or financial relationships that could be construed as a potential conflict of interest.

## Appendix

### Data preparation

Data needs to prepared in long format, with the first row being the variable names and each subsequent row representing a separate measurement. An example data file (“example.csv”) is available with lmmgui. Variable names should begin with a letter, and comprise standard alphanumeric characters (a–z and 0–9)—no spaces or special characters. Implicitly coded nested factors need to be explicitly recoded. For example, consider that in the hypothetical experiment, there was an additional random factor of “Town” because the participants were sampled from different towns. If the listeners from town A are labeled L1, L2… etc., but a different set of listeners from town B are also labeled L1, L2… etc., then the factor listener is implicitly nested in town, and would need to be explicitly recoded, for example as AL1, AL2….BL1, BL2, etc. Next, the data should be saved in text file using the comma (,) or semi-colon (;) as delimiter. In many spreadsheet programs this is achieved by saving in the “.csv” format. The file name should also begin with a letter and comprise standard alphanumeric characters.

### Software prerequisites

Users need to have already installed R, which is available at (www.r-project.org). Next, LMMgui can be downloaded from http://doi.org/10.25592/lmmgui. At present, LMMgui is only available on the Windows platform. If needed, LMMgui will automatically download the R package *lme4*. To visualize the diagnostic plots, a pdf reader is required.

### Output files

Once analysis is complete, a number of text files will be written to the directory of the prepared data. These files allow the user to inspect all the stages of the analysis, including intended analysis steps in R (“.R” files), the actual steps carried out (“.Rout” file), diagnostic plots (“.pdf”) and details about any warnings, if present (“.Warning.txt”).
