# Corrigendum: Linear mixed-effects models for within-participant psychology experiments: an introductory tutorial and free, graphical user interface (LMMgui)

**DOI:** 10.3389/fpsyg.2019.00489

**Published:** 2019-03-12

**Authors:** David A. Magezi

**Affiliations:** Neurology Unit, Laboratory for Cognitive and Neurological Sciences, Department of Medicine, Faculty of Science, University of Fribourg, Fribourg, Switzerland

**Keywords:** linear mixed-effects models, experimental psychology, within-participant design, graphical user interface, R

In the original article there is an error. The link provided in page 1 and the Appendix, for readers to access the software described in the article, is no longer valid.

The old link “http://www.unifr.ch/neurology/en/lmmgui” has now been updated to “http://doi.org/10.25592/lmmgui.”

The author apologizes for this error and states that this does not change the scientific conclusions of the article in any way. The original article has been updated.

